# Investigation of the *miR16-1* (C > T) + 7 Substitution in Seven Different Types of Cancer from Three Ethnic Groups

**DOI:** 10.1155/2009/827532

**Published:** 2009-10-26

**Authors:** Hulya Yazici, Jennifer Zipprich, Tao Peng, Elif Z. Akisik, Hulya Tigli, Mustafa Isin, Ebru E. Akisik, Mary Beth Terry, Ruby T. Senie, Lequn Li, Minhao Peng, Zhiming Liu, Nejat Dalay, Regina M. Santella

**Affiliations:** ^1^Department of Environmental Health Sciences, Mailman School of Public Health, Columbia University, NewYork, NY 10032, USA; ^2^Department of Basic Oncology, Oncology Institute, University of Istanbul, 34452 Beyazit Istanbul, Turkey; ^3^Department of Hepatobiliary Surgery, First Affiliated Hospital of Guangxi Medical University, Gnangxi 530021, China; ^4^Department of Epidemiology, Mailman School of Public Health, Columbia University, NewYork, NY 10032, USA

## Abstract

*Background*. MicroRNAs are a type of small noncoding RNA molecules that have been shown to control gene expression in eukaryotes. Aberrant expression and alteration of miRNAs may be responsible for human diseases including cancer. An *miR16-1* (C > T) + 7 gene mutation has been previously found in familial chronic lymphocytic leukemia patients, one of which reported a family history of breast cancer. *miR16-1* regulates the expression of *bcl-2*, which is important in retinoblastoma, and is located in a genomic region that is frequently lost in nasopharyngeal and hepatocellular carcinomas (HCCs). Therefore, *miR16-1* may be potentially important in the etiology of several solid tumors. To understand the power of the *miR16-1* (C > T) + 7 mutation as a prognostic and diagnostic risk factor, we investigated the mutation in patients with seven different types of cancer including 188 with breast, 102 with ovarian, and 22 nasopharyngeal carcinomas, 96 HCC, 872 chronic myeloid leukemia (CML), 39 chronic lymphocytic leukemia (CLL), and 46 retinoblastoma cases from three different ethnic groups and of hereditary and sporadic etiology. *Methods*. 5′Nuclease TaqMan SNP genotyping assay was used to detect the miR16-1 gene C > T substitution. *Results*. The miR16-1 (C > T) + 7 substitution was not detected in any of the groups studied. *Conclusions*. Considering the large scale of our study, the representation of different ethnicities and levels of hereditary risk, we conclude that the *miR-16-1* (C > T) + 7 mutation is not a good diagnostic or prognostic indicator of risk for the cancers tested.

## 1. Introduction

MicroRNAs are a group of small noncoding RNA (ncRNA) molecules that have been identified in many organisms [1, 2] and shown to regulate the expression of genes in a variety of eukaryotic systems. A recent analysis of the genomic location of human microRNA genes suggested that 50% are located in cancer-associated genomic regions or fragile sites [3, 4]. Recently, Calin et al. identified mutations in six microRNA genes (miR-16-1, miR-27b, miR-29b2/29a, miR122a, miR-187, miR-206) in patients with Chronic Lymphocytic Leukemia (CLL) [5]; none of these mutations were found in a set of 160 individuals without cancer (*P* < .0001) [5]. One germline mutation, miR16-1 C > T + 7, resulted in reduced expression of *miR-16-1* both in vitro and in vivo. Since miR16-1 induces apoptosis by repressing *bcl-2*, a decrease in miR16-1 will result in bcl-2 overexpression, which is a common feature in CLL [6]. Previous data indicated that *miR16-1* and *miR15a* behave as tumor suppressors in CLL. The combination of loss of heterozygosity plus a germline mutation in this region is consistent with the Knudson Model of inactivation of a tumor suppressor gene. Thus, the presence of deleterious mutations in *miR-15a* and *miR-16-1* suggests that this new class of RNAs may play a significant role in many sporadic and hereditary cancers. *miR-15a* and *miR-16-1* are located on chromosome 13q14.3 close to *BRCA2*, which is frequently mutated in hereditary breast and ovarian cancers, and *Rb*, which is deleted or mutated in retinoblastoma and sarcomas. In fact, one of the individuals with CLL in which the mutation was identified reported a family history of breast cancer. Perhaps the mutation in miR16-1 can interact with inactivation of miR16-1 to modify the clinical phenotype [7]. Several cancers, including nasopharyngeal and HCC, have a high frequency of LOH near miR15a/miR16-1. Shao et al. found that 78% of nasopharyngeal tumors had LOH at 13q [8]; another group found that the highest frequency of LOH in nasopharyngeal cancer was at loci D13S133 (53.6%) on 13q14.3 [9]. In HCC, LOH at subchromosomal regions 13q12.3-14.1 and 13q32 was significantly associated with advanced tumor stage and larger tumor size [10]. Further, homozygous deletion of 13q12.11 was significantly associated with early onset HCC [11]. Finally, since there are no data on the miR 16 C > T + 7 mutation in CML, these patients were included in this study. We genotyped 1428 biological samples including tissue and blood DNA from a total of 1365 patients diagnosed with different types of cancer, including breast and ovarian cancers, HCC, CML, CLL nasopharyngeal carcinoma, and retinoblastoma using a TaqMan assay for the *miR16-1* gene C > T substitution.

## 2. Materials and Methods

The study population consisted of 188 breast carcinomas (108 cases without and 80 cases with a family history of breast and ovarian cancer), 102 ovarian cancer (23 cases without and 79 cases with a family history of breast or ovarian cancer), 96 HCC, 22 nasopharyngeal carcinoma, 872 CML, 39 CLL, and 46 sporadic retinoblastoma cases. For some sporadic breast and ovarian cases both blood and tissue DNA was tested (40 of 108 sporadic breast and 23 of 23 sporadic ovarian cancer patients) for a total of 1428 specimens belonging to 1365 cases (Table 1). The HCC cases were recruited between December, 1998 and July, 2005 from an HCC endemic region in southern China. Tissue specimens from ovarian, and nasopharyngeal carcinomas and retinoblastoma patients and blood samples from CML and CLL patients were collected from Turkish patients in the Oncology Institute, University of Istanbul between 1991 and 2000. Samples from breast carcinoma cases were collected in two independent studies; patients were of Turkish (Oncology Institute) and American origin (Columbia University Medical Center) and collected between 1991–2005 and 1995–2005, respectively. Analysis of the *miR16-1* gene C > T substitution was performed using the 5′ nuclease TaqMan SNP genotyping assay. TaqMan primers and probes were designed by Applied Biosystems Inc. (ABI, Foster City, Calif, USA) with sequence data from the Sanger Center Website (http://www.sanger.ac.uk/Software/Rfam/mirna/). The probes were labeled with either FAM or VIC; allele a specific hybridization and degradation allowed for discrimination of the mutated versus the wild type sequence. As a control, two 100 bp synthetic oligonucleotides (Invitrogen, Carisbad, Calif, USA) corresponding to either the wild type or mutant sequence were used to verify the specificity of the probes. The reactions were prepared as follows: 10 *μ*L of TaqMan Universal Master Mix, in 20 *μ*L final reaction volumes, with 20 ng DNA, 0.2 *μ*L TaqMan SNP Genotyping Assay Mix (80X) resulting in a final concentration of 900 nmol/L for the primers, and 200 nmol/L of the probes. Thermal cycling conditions were 95°C for 10 minutes, and then 40 cycles of 95°C for 15 seconds and 60°C for 1 minute. Thermal cycling was performed on an ABI-Realtime 7500 system using the absolute quantitation program standard with ABI SDS software version 1.2.3. Each 96-well plate contained 92 test samples and four reaction controls, including nontemplate, synthetic oligonucleotide homozygous for the C, or T allele and synthetic oligonucleotides combined to make a heterozygote.

## 3. Results and Discussion

MicroRNAs can be involved in cancer initiation and progression and their expression profiling can be exploited for the classification, diagnosis, and prognosis of human malignancies. In addition, germline and somatic mutations in several miRNAs seem to contribute to cancer pre-disposition and progression. To understand this, we searched 1428 biological specimens including tissue and blood DNA from a total of 1365 patients diagnosed with different types of cancer, including breast and ovarian cancers, HCC, CML, CLL, nasopharyngeal carcinoma and retinoblastoma for the *miR16-1* (C > T) + 7 mutation.

We did not detect any mutations in the 1428 samples from 1365 cases analyzed. Our study encompassed a wide range of cancer types including solid tumors (breast, ovarian, nasopharyngeal and HCC), leukemias (CML, CLL) and a childhood cancer (retinoblastoma). We also analyzed DNA samples from individuals representing several ethnicities including American (primarily Caucasian), Chinese and Turkish, and in some cases' (breast and ovarian) individuals with and without a family history of cancer.

The germline mutation found in the *miR-16-1* and *miR15a* primary precursor caused low levels of microRNA expression in vitro and in vivo and was associated with deletion of the normal allele in CLL [5]. However, results have not been consistent [12]. MicroRNAs *miR-15a* and *miR-16-1* are located in the region of a deletion at 13q13.4. This region is frequently deleted or downregulated in CLL cells and the *miR16-1* (C > T) + 7 germ line mutation was found in one of the familial CLL patients who also had a family history of breast cancer [5]. *BRCA2* and *Rb*, mutated in hereditary breast and ovarian cancer and retinoblastoma patients, are relatively close to the *miR16-1 gene*, and some tumors such as nasopharyngeal and HCC have some LOH in the region suggesting that the miR16-1 C > T + 7 mutation may be important in other cancers. A miR16-1 mutation in the 3′ flanking region was recently identified in NZB mice that serve as a model for human CLL, further supporting a unique role for miR16-1 in CLL [13], and it was shown that the C > T substitution on miR16-1 gene affects the level of expression of mature microRNAs [5]. The percentage of mutation in CLL was 2.6% in the study of Calin et al. and one of the patients was a hereditary CLL case and had a familial history of breast cancer history. Moreover, we did not identified any miR16-1 C > T + 7 mutation in our CLL subgroup of cases who had no hereditary CLL and cancer history in their family. Our results for miR16-1 C > T + 7 mutation in CLL are concordant with the study of Borkhardt et al. The frequency of mutation can be different for a particular population and hereditary background of patients and ethnicity can affect the results.

Our results suggest that mutation of miRNA 16-1 is not informative as a prognostic and diagnostic risk factor for the different types of cancers we studied. miR16-1 C > T + 7 mutation may be important in other solid tumors such as colorectal and lung cancer and especially B-cell Non Hodgkin lymphoma which arise from the same progenitor branch of cell lineage as CLL.

## 4. Conclusions

In our large scale study with representation from different ethnicities and with various levels of hereditary risk, we did not identify any cases with the miR-16-1 (C > T) + 7 mutation in any of the cancers tested. However, we tested for the presence of only one type of mutation in one miRNA gene. Many miRNA genes have been investigated for different type of mutations in different type of cancer. Therefore, further research is necessary to determine the importance of the distribution of miRNA mutations in carcinogenesis.

## Figures and Tables

**Table 1:** Distribution of cancer patients in the study.

	Type of cancer	Number of patients	Number of specimen	Source of DNA		Nationality of patients
				Tissue	Blood	
Solid Tumors	Breast Cancer (sporadic)	108	148	108	40	Turkish^§^
	Breast Cancer (hereditary)	80	80	NA	80	American*
	Ovarian Cancer (sporadic)	23	46	23	23	Turkish^§^
	Ovarian Cancer (hereditary)	79	79	NA	79	Turkish^§^
	HCC (Sporadic)	96	96	96	NA	Chinese
	Nasopharyngeal Carcinoma (sporadic)	22	22	22	ND	Turkish^§^

Leukemias	CML	872	872	—	872	Turkish^§^
	CLL	39	39		39	Turkish^§^

Childhood Cancers	Retinoblastoma (Sporadic )	46	46	46	ND	Turkish^§^

Total		1365	1428	295	1134	

*42 Caucasian, 34 Hispanic, 2 Asian, 2 other, ^§^Caucausian NA; material not avaible, ND; not detected.
